# Interstitial Brachytherapy for Orbital Sebaceous Carcinoma

**DOI:** 10.1097/IOP.0000000000002031

**Published:** 2021-07-27

**Authors:** Feng Li, Robert D. Stewart, Paul T. Finger

**Affiliations:** * The Department of Ocular Tumor, Orbital Disease and Ophthalmic Radiation Therapy, The New York Eye Cancer Center, New York, New York, U.S.A.; † The Departments of Radiation Oncology and Ophthalmology, The New York Eye and Ear Infirmary of Mount Sinai, New York, New York, U.S.A.

## Abstract

Sebaceous carcinoma is characterized by its aggressive local tumor behavior and ability to metastasize. Small periocular sebaceous carcinoma are typically treated by excision with cryotherapy. Larger tumors often require adjuvant external beam radiotherapy (EBRT) and/or exenteration surgery. When used alone, EBRT techniques typically exceed the tolerance of critical normal ocular structures. The interstitial orbital brachytherapy-boost technique permits dose escalation to the tumor bed, while minimizing radiation dose to critical normal ocular structures. Here, we present a case of orbital sebaceous carcinoma treated with excision, cryotherapy, and super-thick amniotic membrane fornix reconstruction. Then, after 3 weeks of healing, adjuvant-combined electron interstitial high-dose rate brachytherapy-boost was added to electron-beam radiotherapy to optimize the orbital radiation dose distribution, increase dose to inferonasal orbit, and allow relative sparing of orbital tissues. At 1-year follow-up, there was no evidence of orbital tumor, no significant eye lash loss, normal ocular motility, no radiation retinopathy, optic neuropathy and a visual acuity of 20/20.

## Abstract 2

Here, the authors describe a technique using adjuvant interstitial high-dose rate brachytherapy followed by electron-based external beam radiation therapy to increase the dose to a sebaceous carcinoma tumor bed while reducing dose to normal orbital structures.

## CASE PRESENTATION

**FIG. 1.**
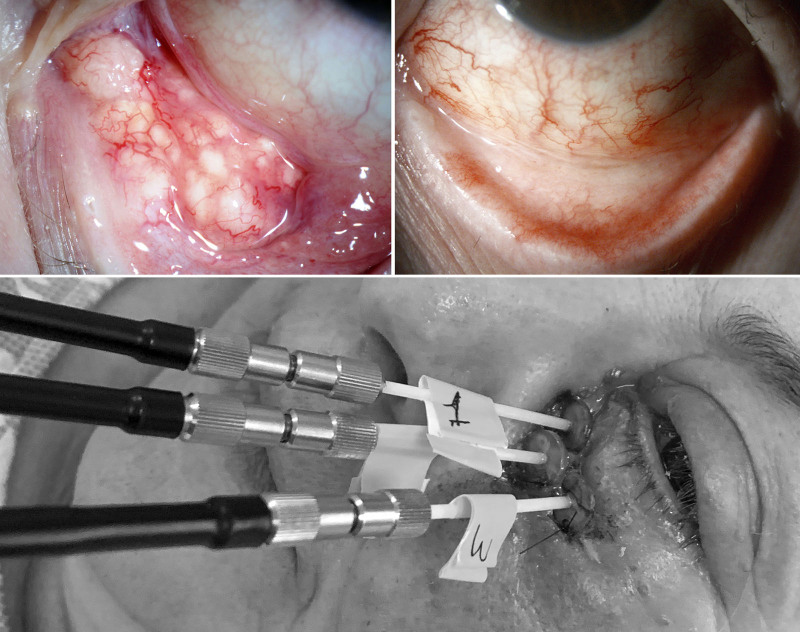
Top left, slit lamp examination showing the multinodular white mass visible in the inferonasal conjunctival fornix. Top right, 1 year following (super-thick amniotic membrane graft) implantation and brachytherapy boost treatment depicting successful epithelialization of the ocular surface and no indications of local recurrence at the conjunctiva. Bottom, external photograph reveals 3 orbital brachytherapy catheters when attached to 3 high-dose rate (HDR) after-loader portals for treatment.

Patient care and reporting conformed to both the Declaration of Helsinki and the Health Information Privacy and Portability Act of 1993 and written consent was obtained from the patient to disclose and publish her medical information. A 67-year-old Caucasian presented with a 2-week history of a rapidly enlarging amelanotic mass in the left medial fornix (Fig. 1, left). She had no history of prior cancer, no ocular motility disturbance and 20/20 vision in OU. Slit lamp examination revealed a multilobular elevated hyper-vascular mass, displacing the lower eyelid. It appeared to be displacing the conjunctiva along its temporal margins (Fig. 1, left). The patient reported no pain on palpation of the relatively hard tumor.

Orbital MRI showed a 1.4 × 1.3 cm mass with its epicenter in the left nasolacrimal fossa, radiographically well-defined margins and no evidence of posterior orbital or bone invasion.

Surgery consisted of the minimally invasive Finger’s Aspiration Cutter Technique biopsy, a diagnosis of carcinoma leading to a request for tissue for extended analysis.^1^ During excision and cryotherapy, the tumor was found to consume the inner lamella of the left lower eyelid, conjunctival fornix and extended into the orbit. Carefully dissected from the surrounding tissues, the tumor was grasped with a large “Finger-tip” cryoprobe and thus elevated to sever its posterior attachments.^2^ Despite its being a nonencapsulated tumor, there was no visible or palpable residual tumor after resection. After tumor margins were treated with cryotherapy, forniceal reconstruction was performed to reconstruct the fornix and prevent symblepharon.^3,4^ Therefore, a 20 × 25 mm super-thick amniotic membrane was free-hand trimmed, inserted into the surgical defect, and sewn into place with both interrupted and running 7–0 Vicryl sutures.^4^

Map biopsies of clinically uninvolved conjunctival tissues were not performed due to our being reasonably certain all the conjunctiva would need be irradiated if the pathologic assessment confirmed that the tumor was sebaceous carcinoma. Final pathologic assessment revealed an unencapsulated American Joint Committee on Cancer 8th edition, T2bN0M0 sebaceous carcinoma with positive margins. Postoperative, total body positron-emission tomography/CT revealed no residual orbital carcinoma or systemic disease.^5^ In an effort to treat presumed orbital microscopic disease, adjuvant radiation therapy was advised. The risks of potential benefits of sole external beam radiotherapy (EBRT) and combination brachytherapy-boost technique were discussed.^6–8^

## RADIATION TREATMENT

**FIG. 2.**
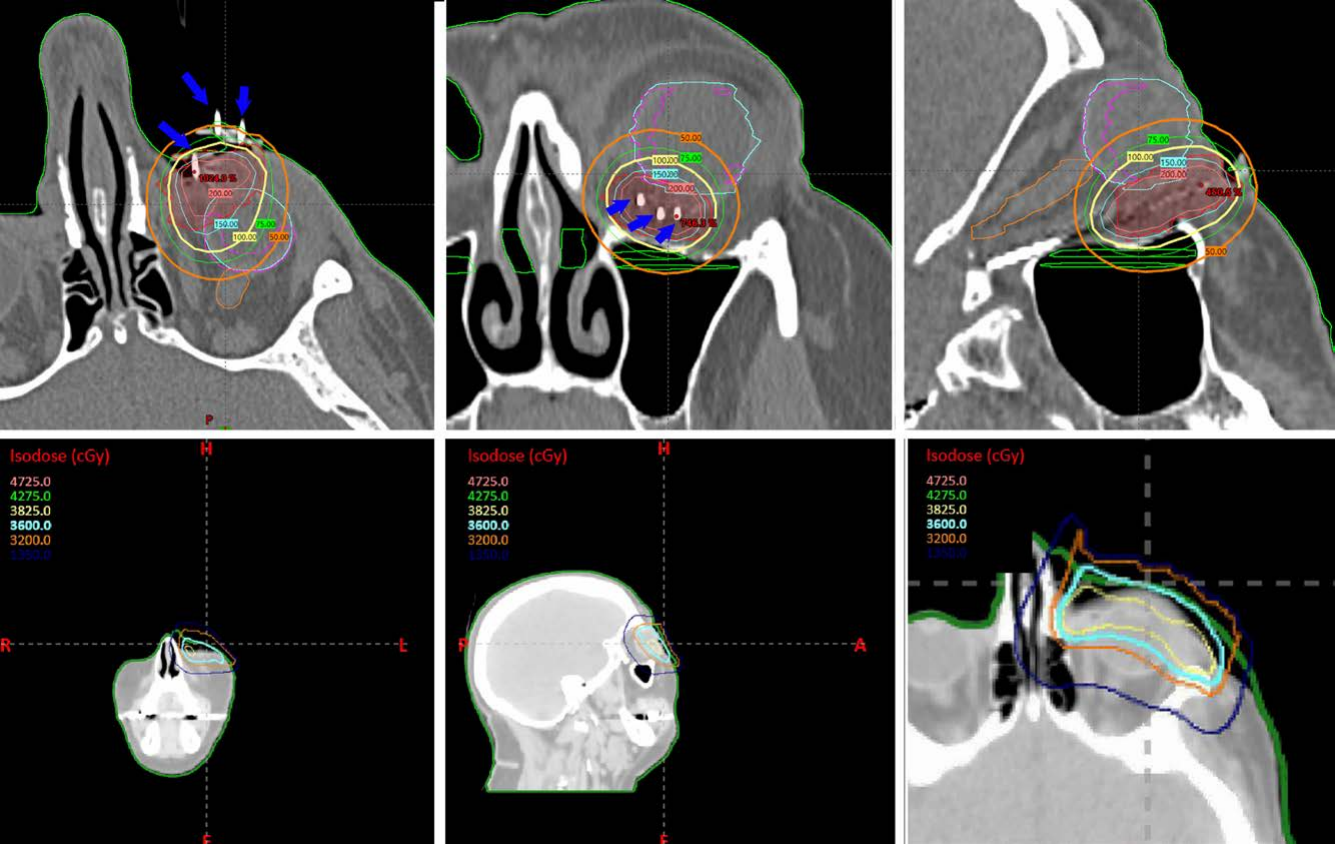
Top, computed axial tomography shows placement of orbital brachytherapy catheters (blue arrows) and surrounding isodose curves depict the iridium-192 “brachy-boost” dose volume. Bottom, computed axial tomography with electron-based EBRT overlay (lower right), a focused transverse view of EBRT field and dose-key. EBRT, external beam radiotherapy.

The radiation treatment plan was created to maximize radiation dose to the tumor bed with relative sparing of lower risk orbital tissues. Our orbital brachytherapy technique has been described.^8^ In sum 3 percutaneous catheters were placed along the inferior orbital rim in the meridians of the primary tumor (Fig. 1, bottom). Each catheter was separated by 1 cm of skin, advanced 4 cm into the orbit and held in place using brachytherapy buttons sutured adjacent to the skin. HDR was delivered using a remote after loader and iridium-192 source. A total 2000 cGy was delivered in five bid 400 cGy fractions, separated by a minimum of 6 hours. Once completed, catheters were removed under peribulbar xylocaine anesthesia (Fig. 2, top).^9^ One month later, 36 Gy in 180 cGy daily fractions using a 9 MeV en face electron field with a custom bolus cutout was delivered to the anterior orbital structures (Fig. 2, bottom). The HDR brachytherapy combined with EBRT for total cumulative dose of 56 Gy.

One year after irradiation our patient’s visual acuity was 20/20 and dry eye treatable with tear supplements (Fig. 1, right). There was no significant keratopathy, iris neovascularization, cataract, radiation retinopathy, or optic neuropathy. However, despite complete local control she developed a sebaceous carcinoma positive cervical lymph node treated with supraomohyoid dissection without adjuvant radiation therapy.

## DISCUSSION

This case demonstrates that interstitial brachytherapy with overlay EBRT can be used to treat select cases of orbital sebaceous carcinoma. Compared with 6 MV or electron-based EBRT alone, brachy-boost increases irradiation of the tumor bed along with relative sparing of surrounding critical structures. This offers a method to preserve the eye and vision. The use of super-thick amniotic membrane forniceal reconstruction was used to prevent symblepharon.^4^

Sebaceous gland carcinoma is characterized by aggressive local behavior, skip lesions, regional nodal, and distant metastasis.^7,8^ Thus, primary local excision is considered insufficient to effect control, due to residual disease and/or microscopic spread. Hence, radiation therapy has been used to extend surgical margins.^5,10^ Orbital sebaceous carcinomas have been treated with EBRT doses of 45–63 Gy that typically exceed the tolerance of critical normal ocular structures leading to severe dry eye, conjunctival keratinization, keratopathy, cataracts, optic neuropathy, retinopathy, and loss of vision. Brachytherapy boost offers a method to improve orbital radiation dose distribution, increase the dose to the tumor bed while reducing dose to critical normal ocular structures. Electron-based EBRT limits treatment to the posterior orbit (optic nerve and macula). Strege et al. reported in a study of 10 children with refractory orbital rhabdomyosarcomas that intensity-modulated brachytherapy using a remote-after loading system improved local tumor control and functional outcomes by allowing for application of various intensities of radiation.^11^ In a retrospective analysis of the results of a clinical case series of patients who underwent postenucleation HDR interstitial brachytherapy, Finger et al. reported 9 patients who tolerated a target dose of 32.85 Gy delivered in 9–10 twice daily fractions. At a median follow-up of 18 months (range, 1–62 months), there was no orbital recurrence and no significant acute or long-term radiation side effects.^9^ A case report by Tagakawa et al. showed that high-dose rate interstitial brachytherapy (HDR-ISBT) of 54 Gy in 9 fractions for a bulky 4.5 × 2.9 × 2.5 cm sebaceous carcinoma of the upper eyelid achieved excellent locoregional control at 18 months after HDR-ISBT.^12^ The present case demonstrates how tailored orbital radiation dose delivery can result in local disease control, functional outcomes, and excellent cosmesis.
